# Systematic review and meta-analysis of antioxidant treatment in patients with acute mountain sickness induced by high altitude exposure

**DOI:** 10.3389/fphys.2026.1779008

**Published:** 2026-03-09

**Authors:** Eduardo Pena, Alexandra Del Río, Sergio Flores

**Affiliations:** 1 High Altitude Medicine Research Center, Arturo Prat University, Iquique, Chile; 2 Facultad de Ciencias de la Salud, Universidad Autónoma de Chile, Temuco, Chile

**Keywords:** acute mountain sickness, antioxidants, high altitude, hypobaric hypoxia, oxidative stress

## Abstract

Acute Mountain Sickness (AMS) is a frequent condition triggered by exposure to hypobaric hypoxia at high altitude. Its pathophysiology involves endothelial dysfunction and oxidative stress, with elevated oxidative molecules identified as key contributors to its development. Antioxidant therapies such as vitamins C/E, α-lipoic acid, and *Ginkgo biloba* have been proposed, though results across studies remain inconsistent.

**Objective:**

This study aims to evaluate the effects of antioxidant treatments in subjects with AMS induced by high-altitude exposure, examining their impact on clinical outcomes and oxidative stress markers.

**Methods:**

A systematic review and meta-analysis were performed according to PRISMA2020 guidelines. Searches were conducted in PubMed, Scopus, and Web of Science through November 2025, supplemented by snowball methods, focusing on studies investigating antioxidant treatments in humans exposed to hypobaric or high-altitude hypoxia. Inclusion criteria were original research in English with full-text availability, human exposure to hypobaric hypoxia, AMS assessment using the Lake Louise Score (LLS), and explicit antioxidant interventions compared with placebo or control. A random-effects meta-analysis using the REML estimator was applied to calculate relative risk (RR), including continuity corrections for zero-event studies. Data extraction was performed in duplicate, and risk of bias was evaluated using the Cochrane tool.

**Results:**

The search yielded 727 records; nineteen studies were included in the qualitative synthesis, and four trials provided comparable dichotomous data for quantitative analysis. Pooled estimates showed a non-significant trend toward reduced AMS incidence with antioxidant treatment (RR ≈ 0.73; 95% CI: 0.47–1.11; p = 0.14). Moderate heterogeneity was detected (I^2^ = 52%, Q p = 0.048). Although not statistically significant, all studies showed a direction of effect favoring antioxidants. Nevertheless, interpretation is limited using pre-2018 LLS diagnostic criteria, absence of studies under chronic intermittent hypobaric hypoxia, and methodological variability.

**Conclusion:**

Current evidence does not demonstrate a statistically significant protective effect of antioxidant therapy against AMS; however, findings remain inconclusive due to few available trials, small sample sizes, pharmacokinetics and pharmacodynamics analysis, and methodological heterogeneity. Larger, well-designed trials with standardized ascent profiles and redox biomarkers are required to determine clinical efficacy.

**Systematic Review Registration:**

https://www.crd.york.ac.uk/PROSPERO/view/CRD420261331390, identifier CRD420261331390.

## 1 Introduction

Acute Mountain Sickness (AMS) is a common and potentially debilitating condition that affects individuals ascending to altitudes above 2,500 m above sea level (Araneda et al., 2005), resulting from a decrease in the partial pressure of gases, particularly oxygen (O_2_), a condition known as hypobaric hypoxia (Bailey and Davies, 2001). AMS manifests with symptoms such as headache, nausea, dizziness, fatigue, weakness, sleep disturbances, and gastrointestinal disorders, typically appearing within 6–12 h after ascent and peaking between 24 and 48 h (Bailey and Davies, 2001; Moraga et al., 2007). Severe AMS, however, is associated with the development of other high-altitude illnesses, such as high-altitude cerebral edema (HACE) and/or high-altitude pulmonary edema (HAPE), both potentially life-threatening and characterized by vasogenic edema, altered vascular permeability, and neurological or respiratory dysfunction (Bailey et al., 2009). Over the years, the prevalence of AMS has increased considerably, as it has been determined that not only mountaineers or climbers are affected, but also individuals working under these conditions in rotational shifts between high-altitude environments and sea level, involving activities such as mining, border security, healthcare, and tourism (Moraga et al., 2007; Alcantara-Zapata et al., 2021). Despite the intermittent exposure system, commonly referred to as chronic intermittent hypobaric hypoxia, being long-term, AMS remains prevalent at the beginning of each exposure cycle in non-acclimatized individuals (Alcantara-Zapata et al., 2021).

Notably, studies have established that exposure to hypobaric hypoxia promotes a redox imbalance, leading to an exacerbated increase in reactive oxygen species (ROS) along with deterioration of endogenous antioxidant defenses (Bailey and Davies, 2001), thereby generating oxidative stress. At the cerebral level, oxidative stress induces alterations in vascular endothelium and blood–brain barrier integrity, affecting capillary permeability and contributing to AMS (Bailey and Davies, 2001; Hefti et al., 2016). This evidence highlights oxidative stress as a key pathophysiological factor in AMS. Several studies have documented significant increases in biomarkers such as malondialdehyde (MDA) and hydrogen peroxide (H_2_O_2_) in exhaled breath condensate (EBC), both at rest and during exercise at altitude, correlating with decreased arterial oxygen saturation and AMS symptom severity (Araneda et al., 2005).

Based on these findings, various studies have proposed antioxidant use as a preventive therapeutic strategy, given that the only pharmacological treatment currently employed to mitigate AMS is acetazolamide, along with anti-inflammatory agents such as dexamethasone and ibuprofen. However, these are indicated only for acute exposure, and acetazolamide presents adverse effects such as paresthesia, dysgeusia, hyperglycemia, and rebound symptoms, limiting its long-term use (Gertsch et al., 2002).

Consequently, studies have evaluated antioxidant compounds such as vitamins C and E, α-lipoic acid, and more recently standardized *Ginkgo biloba* extract, which has been attributed to potential antioxidant effects. In addition, the available evidence suggests an effect in reducing the incidence and severity of AMS in humans (Moraga et al., 2007; Gertsch et al., 2002). However, more studies that report the antioxidant beneficial effects under specific ascent models through meta-analysis are necessary. Based on this evidence, the objective of this systematic review of a meta-analysis is to determine the effects of antioxidant treatments in individuals with AMS, evaluating their impact on Lake Louise Score questionary.

## 2 Methodology

### 2.1 Study design

**FIGURE 1:**
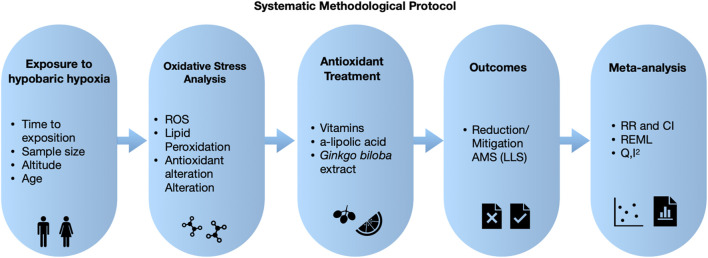
Methodological Planning. Schematic diagram summarizing the methodological protocol for this systematic review and meta-analysis, in accordance with PRISMA 2020 guidelines. It details the key stages of the process, including formulation of the research question using the PICOT framework, database search strategies, study selection process, quality assessment, and qualitative and quantitative synthesis of results; Reactive Oxygen Species (ROS); Reduction/Mitigation of Acute Mountain Sickness (AMS/LLS); Relative Risk and Confidence Interval (RR and CI); Random-Effects Model using Restricted Maximum Likelihood (REML); Cochran's Q and I-squared (Q, I^2^).

The research design followed the PRISMA 2020 protocol for systematic reviews and meta-analyses, aiming to identify studies evaluating the effect of antioxidant therapies on Acute Mountain Sickness, a condition resulting from high-altitude exposure (Figure 1).

### 2.2 Search strategy

A systematic search of scientific literature was conducted in the Web of Science (Core Collection), Scopus, and PubMed databases, including studies published up to November 2025 without country restrictions. The search strategy combined controlled vocabulary with specific keywords: (“hypobaric hypoxia” OR “high altitude”) AND (“antioxidant” AND “oxidative stress”) AND (“pulmonary hypertension” OR “acute mountain sickness”). Terms were combined using Boolean operators AND and OR to maximize the sensitivity and specificity of the search. Additionally, the Snowball method -which consisted of manually screening the reference lists of all articles initially identified through the database search, as well as relevant review articles, to identify additional potentially eligible studies not captured in the primary search strategy-was applied to identify studies specifically evaluating antioxidants for etiological treatment and the palliative effect on Acute Mountain Sickness.

### 2.3 Inclusion criteria

Studies were included if they assessed Acute Mountain Sickness (AMS) induced by exposure to hypobaric hypoxia or high altitude in humans, using the Lake Louise Score (LLS) questionnaire and with oxidative stress markers analysis or defined clinical outcomes. Eligible studies comprised original research articles with full-text availability, clinical trials evaluating antioxidant interventions, and those comparing such interventions with conventional pharmacological treatments or untreated control groups.

### 2.4 Exclusion criteria

Studies were excluded if they were of low quality or had missing data; if they assessed the primary condition using questionnaires or metrics other than LLS; if raw data were unavailable; if published in languages other than English; or if conducted in animals, neonates, permanent residents, or natives of high-altitude regions. Case reports, brief communications, studies without full-text access were excluded. Additionally, studies conducted under normobaric hypoxia or involving Obstructive Sleep Apnea (OSA) were excluded.

### 2.5 Data extraction

Data extraction was independently performed by two reviewers (EP and ADR) in accordance with the PRISMA 2020 guidelines. Any discrepancies were resolved by consensus or, if necessary, through consultation with a third reviewer (SF). A standardized data extraction form was used to collect the following information: first author, year of publication, sample size, type and dosage of the antioxidant intervention, duration of treatment, altitude of exposure, physiological variables, oxidative stress biomarkers, and clinical outcomes defined during exposure to hypobaric hypoxia. This procedure ensured the accuracy, consistency, and reproducibility of the extracted data.

**FIGURE 2:**
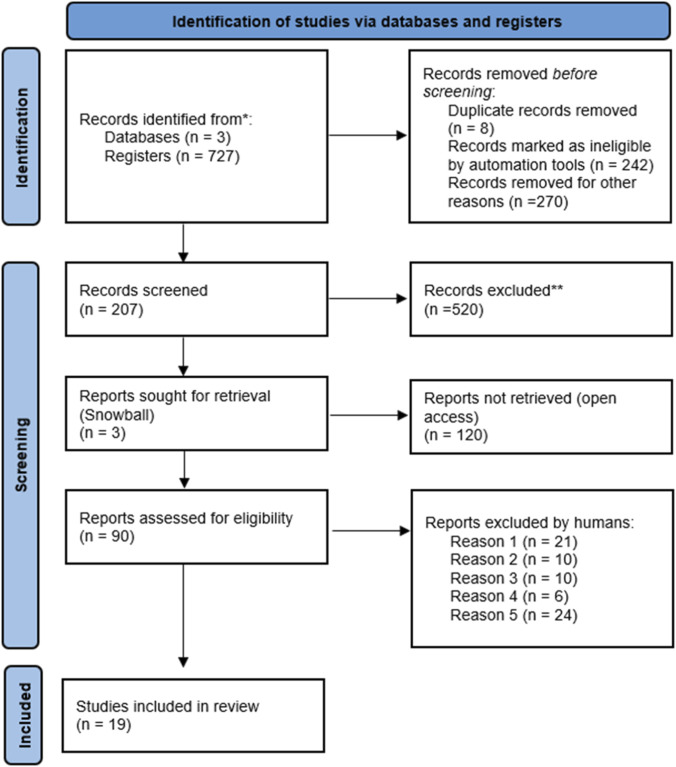
Study Selection Flow Diagram (PRISMA 2020). This figure illustrates the process of identification, screening, eligibility, and inclusion of studies in the review. Studies were excluded for not meeting criteria such as human population, use of LLS assessment, non-hypobaric hypoxia, lack of full text, or absence of relevant clinical data. Nineteen studies were selected for qualitative review, and four studies with comparable dichotomous data were included in the meta-analysis.

As a complement to the database searches, selected references (Moraga et al., 2007; Gertsch et al., 2002; Bailey and Davies, 2001), were included as they met the predefined inclusion criteria. A flow diagram of the study selection process is shown in Figure 2. Most of the included articles followed an uncontrolled before-and-after study design. In total, nineteen studies were included in the qualitative synthesis and four in the quantitative analysis, as not all studies provided sufficient data for meta-analysis.

### 2.6 Quality assessment

Many altitude field studies lacked control groups and were therefore included only in the qualitative synthesis. The quantitative meta-analysis was restricted exclusively to randomized or controlled trials with placebo or untreated comparison groups. Risk of bias was assessed for these trials using the Cochrane Risk of Bias 2 tool, and domains such as randomization, blinding, and incomplete outcome data were considered when interpreting the pooled estimates.

### 2.7 Statistical analysis

For the meta-analysis, only studies reporting comparable dichotomous data on AMS incidence between participants receiving antioxidant treatment and those assigned to placebo or control were included. From the available evidence, only four clinical trials provided the necessary information for quantitative analysis, as they presented 2 × 2 tables with the number of AMS cases in each group according to LLS or equivalent criteria. Individual relative risks (RR) were calculated for each study.

Given differences among trials regarding antioxidant intervention type, altitude reached, exposure duration, and sample size, clinical and methodological heterogeneity was assumed. Therefore, the analysis was performed using a random-effects model with the REML (Restricted Maximum Likelihood) estimator, which provides a more conservative and appropriate pooled effect when variability across studies is expected. The meta-analysis was conducted in R using the metafor package, which computes point estimates of RR and their 95% confidence intervals (CI).

In one trial, the treated group reported no AMS cases, resulting in 0 cells in the 2 × 2 table. To stabilize estimates and enable RR calculation, a continuity correction of 0.5 was applied to all cells, following current methodological recommendations. Heterogeneity was assessed using Cochran's Q statistic and estimates of τ^2^ and I^2^, which quantify the proportion of variability attributable to real differences between studies rather than chance.

Finally, individual effects and the pooled effect were displayed in a Forest Plot, illustrating the direction and magnitude of each trial's effect alongside the overall estimate under the random-effects model.

## 3 Results

The systematic search identified 19 studies evaluating clinical, physiological, or oxidative effects associated with acute or intermittent exposure to hypobaric hypoxia (Table 1). These studies covered a wide range of altitudes, exposure durations, and physiological variables related to AMS and oxidative stress (Figure 2). However, after applying the eligibility criteria, only four clinical trials provided comparable dichotomous data on AMS incidence between an antioxidant-treated group and a control group; therefore, these were the only studies included in the quantitative meta-analysis.

**TABLE 1:** General Characteristics of the Studies Included in the Systematic Review. Summary of the 19 studies that met the criteria for qualitative synthesis in this systematic review.

Author	Year	Altitude (m)	Time exposition	Sample size	Age (years)	Sex	Antiox. treat	Outcomes
Liao et al. (2016)	2016	5.300	5 days	60	21.8 ± 1.8	Male	No	Neutrophil to lymphocyte ratio and acute mountain sickness prevalence after ascent
Strapazzon et al. (2016)	2016	3.830	9, 24, 72 h	16	39 ± 10.2	Male 12/Female 4	No	Optic nerve sheath diameter, ROS production rate, and total antioxidant capacity during acute hypobaric hypoxia
Alcantara-Zapata et al. (2021)	2021	4.350	7 × 7, 4 × 4 for 2 years	338	26 to 60	Male	No	Serum PSA concentration across altitude and hypoxic exposure profiles. Increase Lake Louise Score (4–10 timer first days)
Xu et al. (2025)	2025	3800	7 days	43	26,5 ± 3,17	Male 39/Female 4	No	P300 latency and amplitude (event-related potential), working memory accuracy, and reaction time during exposure to altitude
Hefti et al. (2016)	2016	3.550, 4.590, 6.210	15 days	29	48.8 ± 6.7	Male 21/Female 8	Yes	Circulating endothelial microparticles, plasma 8-isoprostane, and nitric oxide availability under antioxidant vs. placebo at high-altitude
Baillie et al. (2009)	2009	5.200	14 days	83	21.2 ± 2.3	Male 44/Female 39	Yes	Lake Louise score, peripheral oxygen saturation, and incidence of acute mountain sickness under antioxidant or placebo treatment
Araneda et al. (2005)	2005	2.500, 3.000, 4.000, 5.000, 6.125	15 days	10	29 ± 4.3	Male	No	Exhaled breath hydrogen peroxide and malondialdehyde levels during high-altitude ascent and their correlation with acute mountain sickness scores
Julian et al. (2014)	2014	4.875	9 h	20	27.8 ± 2.3	Male 17/Female 3	No	Quantitative proteomic profiles of redox related proteins after hypobaric hypoxia exposure in AMS-susceptible and resistant individuals
Luks et al. (2017)	2017	3.500, 5.300, 5.900	13 days	11	47 ± 12	Male 8/Female 3	No	Peripheral oxygen saturation, ventilatory response, and acute mountain sickness incidence during high-altitude trekking
Bailey et al. (2010)	2010	4559	44 h	38	37 ± 10	Male 32/Female 6	No	Pulmonary artery systolic pressure, free radical-mediated lipid peroxidation, inflammatory and nitrosative stress biomarkers during rapid ascent
Tang et al. (2018)	2018	3.500, 4.300	24 h	261	20.9 ± 2.4	Male	No	Plasma osteopontin, superoxide dismutase, malondialdehyde, and Lake Louise score during acute high-altitude exposure
Heinzer et al. (2016)	2016	3.450	26 h	13	34 ± 9	Male	No	Apnea–hypopnea index, oxygen desaturation index, and minimum nocturnal polysomnography under normobaric and hypobaric hypoxia exposure
Malacrida et al. (2019)	2019	3.830	24 years 72 h	15	39 ± 10.2	Male 11/Female 4	No	ROS production rate, plasma total antioxidant capacity, thiobarbituric acid reactive substances, and optic nerve sheath diameter during acute hypobaric hypoxia exposure
Mellor et al. (2013)	2013	1.300, 3.400, 4.270, 5.150, 5.364	10 days	52	35.5 ± 1.1	NR	No	Plasma neutrophil gelatinase-associated lipocalin concentrations and Lake Louise Score in subjects exposed to altitude
Citherlet et al. (2024)	2024	3.375	14 h	13	32 ± 8	Female	No	Hypoxic ventilatory response, peripheral and cerebral oxygenation, and oxidative stress markers during different phases of the menstrual cycle and their association with AMS after acute exposure to altitude
Bailey and Davies (2001)	2001	5.180	10 days	18	35 ± 10	Male 16/Female 2	Yes	Lake Louise Score, peripheral arterial oxygen saturation, and energy intake under antioxidant supplementation is an effective intervention that improves the physiological profile of AMS.
Li et al. (2011)	2011	3.700	2 h	1.234	17 to 22	Male	No	Mitochondrial DNA haplogroup distribution and Lake Louise score in relation to AMS.
Moraga et al. (2007)	2007	3.696	24 h/3 days	36	22.5 ± 2	Male	Yes	Incidence of acute mountain sickness, arterial oxygen saturation, and heart rate under *Ginkgo biloba*, acetazolamide, or placebo
Gertsch et al. (2002)	2002	2.835, 4.205	24 h	26	28 and 33	Male 12/Female 14	Yes	Lake Louise report score, incidence and severity of acute mountain sickness, and need for descent after rapid ascent with 1-day *Ginkgo biloba* or placebo pretreatment

The table presents data on altitude reached, duration of exposure to hypobaric hypoxia, sample size, antioxidant intervention, and participants' age and sex. All studies included clinical assessment of AMS using the Lake Louise Score (LLS). Among these, only four studies provided comparable dichotomous data on AMS incidence, which were included in the quantitative meta-analysis.

**Prostate-specific antigen (PSA), No reported (NR).

The included trials were Baillie et al. (2009), Bailey and Davies (2001), Bailey et al., 2010, Moraga (2007), Gertsch et al. (2002), all of which evaluated antioxidant interventions, vitamins C and E, α-lipoic acid, or standardized *Ginkgo biloba* extracts, compared with placebo, determining AMS using variations of the LLS (Table 2).

**TABLE 2:** Selected studies included in the meta-analysis (n = 4). Analyzing experimental protocols: geographic altitude, doses, period of administration, time to high altitude exposure and number of participants.

Author	Year	Altitude (m)	Dose	Time exposition	AMS measured	Number participants	Principal results
Baillie et al. (2009)	2009	5,200	Vitamin C (L-ascorbic acid): 1 g/day; Vitamin E (α-tocopherol acetate): 400 IU/day; α-lipoic acid: 600 mg/day	14 days	LLS*	83	SpO2 (decrease ∼77% to 5.200 m. NC: Placebo 75%; Antioxidant 76%), PASP (increase ∼30.34 mmHg to 5.200. NC: Placebo 32 mmHg, Antiox. 33 mmHg), hematocrit (increase ∼44%–∼49%. NC: Placebo 49.9%, Antiox. 48.5%), pCO2 (decrease ∼3.2–3.6 kPa. NC Ambos ∼3.3 kPa), H+ ∼30,4–33.7 mmol/L. NC: ambos ∼33.5 mmol/L), LLS (NC: placebo 4 (IQR 2.6); Antioxidant 5 (IQR 3.7)), VAS (NC: placebo 224 ± 103, Antioxidant 243 ± 116)
Bailey and Davies (2001)	2001	5,180	L-ascorbic acid (1000 mg/day), dl-α-tocopherol acetate (400 IU/day), α-lipoic acid (600 mg/day)	10 days	LLS*	18	Lake Louise score, peripheral arterial oxygen saturation, and energy intake under antioxidant supplementation is an effective intervention that improves the physiological profile of AMS.
Moraga et al. (2007)	2007	3,696	*Ginkgo biloba* (80 mg every 12 h = 160 mg/day): EGb 761 extract, standardized to 24% flavonoids and 6% terpene lactones; placebo	3 days	LLS*	36	Incidence of acute mountain sickness, arterial oxygen saturation, and heart rate under *Ginkgo biloba*, acetazolamide, or placebo
Gertsch et al. (2002)	2002	2.835, 4.205	*Ginkgo biloba* (60 mg every 8 h = 180 mg/day): standardized GK501 extract, containing 24% ginkgoflavonoids and 6% terpenes (ginkgolides A, B, and C)	4 h/2 days	LLS*	26	Lake Louise report score, incidence and severity of acute mountain sickness, and need for descent after rapid ascent with 1-day *Ginkgo biloba* or placebo pretreatment

Optic nerve sheath diameter, ONSD; total antioxidant capacity, TAC; Thiobarbituric acid-reactive substances, TBARS; 8-isoprostanes, 8-isoPGF2α; beats per minute, bpm; Lake Louise Score, LLS; Prostate-Specific Antigen, PSA; Hemoglobin, Hb; no significant difference, NDS; Systolic Blood Pressure, SBP; Diastolic Blood Pressure, DBP; Mean Arterial Pressure, MAP; Capillary blood gases, pCO2; No Changes, NC; Peroxiredoxin 6, PRDX6; Glutathione peroxidase 3, GPX3; Sulfhydryl oxidase 1, QSOX1; Plasma Kallikrein, KLKB1; Vitronectin, VTNC; Factor XIII, F13A; Cholinesterase, CHLE; Complement C8, CO8A; Dimensionless unit, u; Erythropoietin, EPO; Transvalvular Pressure Gradient, TVPG; Everest Base Camp 5.300 m, EBC; Namche Bazaar 3.500 m, NB; Cyclic Guanosine Monophosphate, cGMP; Lipid Hydroperoxides, LOOH; S-nitrosothiols, RSNO; Red Blood Cell-bound Nitric Oxide, RBC-NO; Nitric Oxide, NO; 3-Nitrotyrosine, 3-NT; Pulmonary Artery Systolic Pressure, PASP; Ascorbate Radical, A•−; Heart Rate, HR; Osteopontin, OPN; Superoxide dismutase, SOD; Malondialdehyde, MDA; Heart Rate, HR; Total Sleep Time, TST; Apnea-Hypopnea Index, AHI; Rapid Eye Movement Sleep, REM; Non-Rapid Eye Movement Sleep, NREM; Time spent in deep sleep, N3; Wake After sleep onset, WAKE; Breath Rate, BR; Neutrophil gelatinase-associated lipocalin, NGAL; high-sensitivity C-reactive protein, hsCRP; develops the pathology, AMS+; does not develop it, AMS-; Hypoxic Ventilatory Response, HVR; Malondialdehyde, MDA; Superoxide Dismutase, SOD; Early Follicular Phase, Fol1; Late Follicular Phase, Fol2; Mid-Luteal Phase, Lut3; Minute Ventilation, VE.: studies used pre-2018, versions of the Lake Louise Score.

The random-effects meta-analysis (REML method) showed a non-significant trend toward reduced AMS risk among participants receiving antioxidant treatment, with a log[RR] of −0.32, corresponding to an approximate relative risk of 0.73, suggesting a potential 27% reduction in AMS risk compared to control. However, this reduction did not reach statistical significance (95% CI: 0.47 to 1.11; *p* = 0.14). Despite this, the direction of effect was consistent across all four trials, with no evidence of increased risk in any individual study, supporting the coherence of the observed clinical signal.

Confidence intervals were wide in some studies, particularly those with small sample sizes or zero-event cells, as in Moraga et al. (2007), where no treated subjects developed AMS. To address this, a standard continuity correction (+0.5) was applied, maintaining model stability. Despite these limitations, all trials numerically favored antioxidant treatment.

Heterogeneity analysis showed a I^2^ of 52%, indicating moderate heterogeneity among studies, which is expected given variability in intervention types (vitamins vs. *Ginkgo biloba*), altitude differences, exposure times, and ascent protocols, which are presented in the Table 1. The Q test was significant (Q = 7.89; *p* = 0.048), confirming that part of the variability among studies is not solely due to sampling error.

**FIGURE 3:**
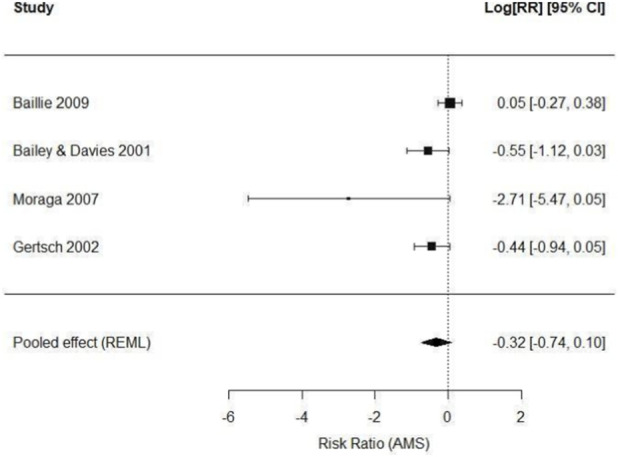
Effect of Antioxidant Interventions on AMS Incidence. Forest Plot of the random-effects meta-analysis showing the risk ratio (RR) and 95% confidence intervals (CI) for AMS incidence in participants receiving antioxidant treatment compared with placebo or control. Although statistical significance was not reached (RR: 0.73; 95% CI: 0.47–1.11; p = 0.14), the direction of effect consistently favored antioxidant intervention. Moderate heterogeneity was observed (I^2^ = 52%).

The quantitative synthesis of the four included trials is presented in Figure 3 (Forest Plot), showing each study's individual contribution to the pooled effect, the consistent direction toward potential clinical benefit, and the width of confidence intervals associated with each estimate.

### 3.1 Risk of bias assessment

Risk of bias was evaluated using the Cochrane RoB 2 tool for the four controlled trials included in the quantitative synthesis. Three studies were judged to present some concerns for risk of bias, primarily related to small sample sizes, incomplete outcome data, or early termination of follow-up (Bailey and Davies, 2001; Gertsch et al., 2002; Baillie et al., 2009). One study was classified as high risk of bias due to the absence of blinding in a trial relying on subjective symptom-based outcomes (Lake Louise Score), increasing the likelihood of performance and detection bias (Moraga et al., 2007). Overall, randomization and allocation procedures were generally adequate across studies. Excluding the high-risk study in sensitivity analyses did not materially change the pooled effect estimate, supporting the robustness of the findings.

### 3.2 Sensitivity analysis

Excluding the study judged to be at high risk of bias (Moraga et al., 2007) produced similar results (RR = 0.77, 95% CI 0.52–1.15) compared with the primary analysis (RR = 0.73, 95% CI 0.48–1.11), with no material change in the direction or interpretation of the pooled effect. Leave-one-out analyses showed consistent effect estimates across all exclusions, with pooled risk ratios ranging from 0.59 to 0.77, and no single study reversing the direction of effect. These findings indicate that the overall results were robust to individual study removal.

## 4 Discussion

This systemic review and meta-analysis compiled the only available studies that directly evaluated the effect of antioxidant interventions on the incidence of AMS under hypobaric hypoxia conditions in human studies. Although the number of included trials was limited, the results showed a consistent trend toward reduced AMS risk among subjects receiving antioxidant treatment, with a pooled effect suggesting an approximate 27% relative risk reduction. This direction of effect aligns with the pathophysiological hypothesis linking oxidative stress to AMS symptom development (e.g., endothelial dysfunction, increase permeability) and the potential benefit of strategies that modulate ROS production or enhance endogenous antioxidant capacity (Siques et al., 2018).

An additional consideration is the methodological quality of the included trials. Most studies presented some concerns for risk of bias related to small sample sizes, incomplete follow-up, or early termination, and one trial lacked blinding while relying on subjective symptom-based outcomes. However, sensitivity analyses excluding this higher-risk study yielded comparable pooled estimates, and leave-one-out analyses showed consistent direction of effect across all exclusions. These findings suggest that the observed trend toward benefit is unlikely to be driven by a single biased study, although the overall certainty of evidence remains limited by imprecision and heterogeneity.

Despite this clinical signal, statistical significance was not reached, likely reflecting limitations in the available evidence. The trials were small, varied in exposure duration and attained altitude, and differed in the type of antioxidant strategies (vitamin-based regimens versus *Ginkgo biloba* extracts), all of which contributed to heterogeneity (I^2^ ≈ 52%). This pattern mirrors the substantial heterogeneity (I^2^ ≈ 58.7%) seen in a dedicated meta-analysis of *Ginkgo biloba* for AMS prophylaxis, which reported a non-significant relative risk reduction overall. Additionally, the vitamin antioxidant literature is mixed: a small, randomized ascent to Everest Base Camp suggested lower Lake Louise scores and higher SpO_2_ with vitamins C/E plus α lipoic acid, whereas a larger placebo-controlled expedition to ∼5200 m found no preventive benefit from oral antioxidants. Together, these findings explain imprecision and wide confidence intervals, especially in trials with zero-event cells (Bailey and Davies, 2001; Baillie et al., 2009; Tsai et al., 2018). Moreover, pharmacokinetic constraints may help explain the heterogeneous clinical findings. For vitamin C, intestinal uptake is saturable, plasma concentrations are tightly regulated, and oral dosing produces modest increases with rapid renal elimination; even large oral doses rarely exceed 220 μmol/L in plasma (Padayatty et al., 2004), which could limit sustained systemic exposure at high altitude. For α-tocopherol (vitamin E), absorption depends on dietary fat and chylomicron transport, with hepatic α-tocopherol transfer protein (α-TTP) determining tissue delivery (Traber, 2007; Mohd Zaffarin et al., 2020); variable bioavailability and formulation differences can blunt expected antioxidant effects *in vivo* α-Lipoic acid (ALA) shows rapid absorption but short half-life and ∼20–40% oral bioavailability, with marked inter-formulation variability; timing and dosing relative to ascent may therefore be critical (Salehi et al., 2019). For *Ginkgo biloba*, PK depends on the specific extract; standardized EGb 761 yields measurable terpene lactones/flavonoids with t½ ∼3–11 h, but composition varies across products, potentially altering exposure and outcomes, consistent with conflicting AMS results when different extracts were used. Collectively, these PK features create mismatches between mechanistic antioxidant promises and clinical efficacy, especially when ascent is rapid and symptom onset precedes peak systemic levels.

It is noteworthy that no individual study showed increased AMS risk with antioxidant use and all point estimates favored treatment. Such directional consistency suggests a potential benefit that cannot be ruled out but likely requires better-designed, adequately powered randomized trials with standardized exposure and clinical endpoints. Future studies should adopt the 2018 Lake Louise AMS Score update, which removed “sleep disturbance” to improve diagnostic specificity for AMS, thereby reducing outcome misclassification in prophylaxis trials. Pre-specifying pharmacologic subgroups (vitamin antioxidants vs. standardized *Ginkgo biloba* preparations vs. other nutraceuticals) could further clarify sources of heterogeneity (Tsai et al., 2018; Roach et al., 2018).

The findings reported (Mohd Zaffarin et al., 2020) support the central hypothesis of this meta-analysis, demonstrating that oxidative stress is a key mechanism in the pathophysiology of AMS and that antioxidant treatments such as astaxanthin (AST) could significantly attenuate its effects. Since, studies with this compound have demonstrated antioxidant and anti-inflammatory protective effects in patients after renal transplant and in the correction of oxidative status in aging individuals (Pena et al., 2024; Fassett et al., 2008). Although, to our knowledge, no human studies have evaluated the use of AST in AMS under hypobaric hypoxia conditions, however, recent studies in animal model under condition particular to hypobaric hypoxia such as chronic intermittent hypobaric hypoxia (CIHH), AST reduced right ventricular hypertrophy and normalized oxidative stress biomarkers such as MDA and Nox2, while increasing glutathione peroxidase activity. These results are consistent with clinical studies included in this review, where antioxidants such as vitamins C and E and *Ginkgo biloba* showed protective effects in humans exposed to high altitude. Although the meta-analysis did not yield statistically significant results, both preclinical and clinical evidence suggest that antioxidant use represents a promising strategy to mitigate the adverse effects of acute high-altitude exposure. However, it is important to consider the kind of exposure to high altitude, since, for example, this research not considerate the CIHH exposition in the meta-analysis, which open a new avenue to the knowledge. Finally, AST is a highly lipophilic carotenoid with low and formulation-dependent oral bioavailability; lipid-based or micellar preparations can enhance exposure, and reported human t½ is ∼16 h after a single oral dose, yet clinical effects depend on reaching adequate target-tissue levels. However, we identified no human studies evaluating AST for AMS under hypobaric hypoxia, and available data are limited to mechanistic/animal contexts; consequently, its discussion has been trimmed and moved to Future Directions, explicitly acknowledging the absence of clinical evidence and the PK uncertainties (dose, timing, BBB penetration, and formulation effects) relevant to rapid ascent scenarios.

From a clinical practice standpoint, it is essential to interpret these signals against current guidelines. The Wilderness Medical Society (WMS) 2024 update and its JAMA synopsis emphasize gradual ascent as first-line prevention and recommend acetazolamide (and dexamethasone in selected circumstances) for pharmacologic prophylaxis; antioxidants are not included among interventions with sufficient evidence. A comprehensive meta-analysis confirms that acetazolamide significantly reduces AMS incidence across multiple dosing regimens (125–375 mg BID), with trial sequential analyses indicating adequate accumulated evidence. Furthermore, emerging strategies, such as acetazolamide combined with remote ischemic preconditioning (RIPC), have shown additional risk reductions under controlled hypoxia exposure. Public health guidance (CDC Yellow Book 2026) likewise prioritizes staged ascent and acetazolamide/dexamethasone, underscoring the current evidence gap for antioxidants as standard prophylaxis (Gao et al., 2021; Derstine and Luks, 2024; Liu et al., 2024; Luks et al., 2024).

In summary, the present results indicate that antioxidants may confer a modest protective effect against AMS and that this effect is physiologically coherent given the contribution of oxidative stress to endothelial dysfunction, vascular permeability, and inflammatory signaling in hypoxia. However, certainty remains limited, and antioxidants cannot be recommended as routine prophylaxis. To move the field forward, we need robust randomized trials under controlled ascent profiles, adequate sample sizes, and standardized endpoints. Incorporating oxidative stress biomarkers (e.g., MDA, Nox2, GPx), clear classification of hypoxia modality (hypobaric vs. normobaric), standardized dosing/purity for botanicals, and prespecified subgroup analyses (vitamins, AST, other nutraceuticals) will be critical to confirm, or refute, the clinical utility of antioxidant interventions for AMS.

### 4.1 Pharmacokinetic and pharmacodynamic considerations of antioxidant supplementation in AMS

As mentioned above, oxidative stress has been proposed as a key contributor to the pathophysiology of AMS, driven by hypobaric hypoxia–induced mitochondrial dysfunction, increased ROS generation, endothelial activation, and neurovascular dysregulation. Based on this biological rationale, several clinical trials have evaluated antioxidant supplementation as a preventive strategy for AMS. However, clinical outcomes have been inconsistent, ranging from apparent benefit to complete lack of efficacy.

This discrepancy highlights a critical mechanism–efficacy gap, where biologically plausible antioxidant mechanisms fail to translate into consistent clinical benefit. A pharmacokinetic (PK) and pharmacodynamic (PD) analysis of the antioxidants used in these trials suggests that many inconclusive results may reflect pharmacokinetic limitations rather than true pharmacodynamic inefficacy.

In this meta-analysis study, we evaluated two main antioxidant strategies, such as vitamin-based antioxidant supplementation (Vitamins C, E, β-Carotene) (Bailey and Davies, 2001; Baillie et al., 2009) and *Ginkgo biloba* extract (Gertsch et al., 2002; Moraga et al., 2007); Where although both approaches target oxidative stress, their PK/PD profiles differ substantially, which may partially explain divergent clinical findings.

For example, respect to vitamin antioxidant supplementation the study by Bailey and Davies (2001), the antioxidant vitamins were administered prophylactically and were associated with a reduction in AMS symptoms. However, this study did not report plasma or another biological sample antioxidant concentration, limiting interpretation of whether therapeutic levels were achieved. Vitamins C and E exhibit highly variable oral bioavailability, influenced by intestinal absorption saturation, first-pass metabolism, and baseline nutritional status, which could be considered as PK limitation. In contrast, Baillie et al. (2009) conducted a well-powered, double-blind randomized controlled trial and found no protective effect of oral antioxidant vitamin supplementation against AMS. Highlighting that this study also did not measure circulation antioxidant concentration, leaving open the possibility that administered doses failed to achieve sufficient systemic exposure to counteract hypoxia-induced ROS production.

Moreover, it is important to consider the half-life, since the vitamin C has a short plasma half-life and is tightly regulated by renal excretion under 24 h (Chen et al., 2022), while vitamin E, although lipophilic, exhibits slow plasma kinetics, reaching peak concentrations only after 18–20 h—indicative of delayed absorption and distribution—while also a slow plasma disappearance rate typical of lipophilic vitamins, thereby necessitating prolonged administration to achieve steady-state tissue levels (Traber et al., 2022). The dosing regimens used in these trials may have been adequate for sea-level antioxidant support but insufficient for the marked increase in oxidative stress associated with rapid ascent or long-intermittent exposure to hypobaric hypoxia conditions. Therefore, the absence of clinical efficacy in Baillie et al. (2009) may reflect subtherapeutic exposure relative to the oxidative burden, rather than a failure of antioxidant mechanisms itself. Finally, is to be considered that the AMS involves central nervous system processes, altered cerebral blood flow, and neuroinflammation. Therefore, antioxidant efficacy depends not only on systemic availability but also on penetration into the central nervous system or blood-brain barrier (BBB). Water-soluble antioxidants such as vitamin C cross the BBB via specific transporters (GLUT and SVCT), but their transport is saturable and tightly regulated (Rivas et al., 2024). On the other hand, lipid-soluble antioxidants as vitamin E cross the BBB slowly and may require prolonged exposure to achieve meaningful brain concentrations, where none of the vitamin-based AMS trials assessed these agents reach the relevant target tissues, this evident limitation strongly constrains interpretation of clinical outcomes and underscores the PK-PD disconnect between theoretical neuroprotective effect and observed efficacy (Table 3).

**TABLE 3:** Pharmacokinetic and pharmacodynamic considerations of antioxidant interventions evaluated for the prevention of AMS.

Study (Year)	Antioxidant intervention	Doses and timing	Reported clinical outcome on AMS	Plasma/Serum levels reported	PK/PD limitations and mechanisms-efficacy gap	PK considerations (Bioavailability/Half-Life)	BBB penetration potential
Baillie et al. (2009)	Vitamins C, E, β-carotene	Oral supplementation prior to and during ascent	Reduced AMS symptom severity	Not reported	Clinical benefit observed, but absence of plasma/tissue concentration data prevents confirmation of adequate antioxidant exposure at target tissues; possible underestimation of PK constraints	Oral bioavailability variable; vitamin C exhibits saturable absorption and rapid renal clearance; vitamin E requires prolonged dosing to reach steady-state tissue levels (Chen et al., 2022; Traber et al., 2022)	Limited; vitamin C transport across BBB is saturable; vitamin E crosses BBB slowly
Bailey and Davies (2001)	Vitamins C, E, β-carotene	Oral supplementation prior to ascent	No preventive effect on AMS	Not reported	Negative outcome may reflect insufficient systemic and CNS exposure rather than lack of antioxidant efficacy; PK failure cannot be excluded	Doses likely adequate for sea-level antioxidant support but possibly insufficient under hypoxic oxidative stress; short half-life of vitamin C (Chen et al., 2022)	Limited and slow BBB penetration
Gertsch et al. (2002)	*Ginkgo biloba* extract	Initiated 1 day before rapid ascent	No reduction in severe AMS	Not reported	Short pre-exposure period likely insufficient to achieve effective PK steady state; timing may explain lack of efficacy	Moderate oral bioavailability: flavonoids and terpenoids require repeated dosing to achieve stable tissue levels (Li et al., 2022; Anastassakis, 2022)	Partial BBB penetration reported for some constituents
Moraga et al. (2007)	*Ginkgo biloba* extract	Oral supplementation before ascent to 3,696 m	Significant reduction in AMS incidence	Not reported	Positive outcome supports PD plausibility; however, lack of PK measurements prevents defining exposure–response relationship	Similar PK profile to other *Ginkgo biloba* extract studies; efficacy may depend on dosing duration and ascent profile (Li et al., 2022; Anastassakis, 2022)	Partial BBB penetration

Acute Mountain Sickness, AMS; Brain Blood Barrier, BBB; Pharmacokinetic, PK; Pharmacodynamic, PD.

Regarding the PK-PD profiles of *Ginkgo biloba* extract, this compound exhibits pleiotropic pharmacodynamic effects, e.g., antioxidant and free radical scavenging activity and anti-platelet activating factor activity (Li et al., 2022). Additionally, vasoregulation effect via nitric oxide modulation, improving microcirculatory flow (Anastassakis, 2022). These combined actions may be particularly relevant in AMS, since as mentioned above, where oxidative stress, endothelial dysfunction, and microvascular dysregulation coexist. However, study by Gertsch et al. (2002) Reported no protective effect of *Ginkgo biloba* against severe AMS when administration began on day prior to rapid ascent. In contrast, Moraga et al. (2007) Observed a significant reduction in AMS Incidence in individuals ascending to 3,696 m in northern Chile, from PK perspective, these discrepancies may reflect differences in the timing of administration relative to ascent, duration of pre-exposure dosing and individual variability in absorption and metabolism of flavonoids and terpenoids. *Ginkgo biloba* constituents have moderate oral bioavailability and undergo hepatic metabolism, with plasma half-life that may require several days of dosing to reach stable pharmacodynamic effects. Initiating supplementations only 1 day prior to ascent, as in Gertsch et al. (2002) may have been insufficient to achieve effective tissue concentrations, particularly in the brain and pulmonary and vasculature.

Then, an often-overlooked factor in these trials is the effect of hypobaric hypoxia itself on supplement disposition, since this environment factor can alter the gastrointestinal absorption due to reduced splanchnic perfusion (McKenna et al., 2022), hepatic metabolism via hypoxia-inducible factor (HIF)-mediated enzyme regulation (Luo et al., 2023) and renal clearance and plasma volume through hemoconcentration (Schlittler et al., 2021), these alterations mentioned can reduce the supplement exposure or alter antioxidant kinetics in unpredictable ways. None of the reviewed trials presented explicitly accounted for hypoxia-induced alteration in PK or/and PD, representing a major limitation in study design and interpretation (Table 3).

Therefore, respect to PK/PD perspective, the lack of consistent efficacy across antioxidant trials in AMS cannot be attributed solely to absence of biological effect. Instead, several alternative explanations emerge, such as: Insufficient dosing relative to oxidative stress burden, short duration of administration prior to hypoxic exposure, failure to reach central nervous system target sites and hypoxia-induced alterations in absorption, distribution, metabolism, and elimination.

Limitations: These findings should be interpreted with caution. All included studies used pre-2018 versions of the Lake Louise Score, which differ from current diagnostic criteria and may affect the reported incidence and severity of acute mountain sickness. In addition, no studies conducted under conditions of chronic intermittent hypobaric hypoxia (CIHH) were identified, limiting the applicability of the results to this exposure pattern. Finally, heterogeneity in ascent profiles, altitude reached, duration of exposure, and supplementation protocols, quantified by the I^2^ statistic and addressed using a random-effects model, may have contributed to variability across studies and constrained further stratified or sensitivity analyses.

### 4.2 Future clinical perspectives

Recent work in the field underscores the need for broader and more integrative approaches to AMS therapeutics, recognizing that oxidative stress, inflammation, and disruptions in energy metabolism interact dynamically during exposure to hypobaric hypoxia. There is growing consensus that antioxidant-based strategies should progress beyond single-agent interventions and instead move toward multi-target approaches capable of simultaneously modulating redox balance, inflammatory pathways, and hypoxia-responsive signaling networks. Emerging evidence also suggests that therapies directed at redox regulation may be more effective when combined with interventions targeting mitochondrial function and key hypoxia-induced molecular mechanisms, highlighting the importance of mechanistically guided clinical trials using standardized biomarkers and altitude-exposure protocols. In parallel, recent developments in ethnopharmacology point to novel therapeutic opportunities based on multi-component botanical formulations that influence hypoxia-related signaling, reduce oxidative burden, and enhance metabolic adaptation.

In addition, the antioxidant trials in AMS should incorporate the measurement of plasma or another sample antioxidant levels, PK-guided dosing strategies tailored to hypobaric hypoxia conditions, consideration of BBB penetration for centrally mediated symptoms and longer pre-acclimatization supplementation periods. Since, without integrating PK/PD principles, clinical outcomes alone are insufficient to validate or refute the therapeutic potential of antioxidant strategies in AMS induced by hypobaric hypoxia conditions. Together, these perspectives indicate that future AMS research should prioritize well-powered, mechanistically informed randomized trials, integration of redox and inflammatory biomarkers, and the exploration of multi-target antioxidant strategies that better reflect the complex pathophysiology of altitude illness.

## 5 Conclusion

Antioxidants may confer a protective effect against the development of AMS by modulating oxidative stress induced by hypobaric hypoxia under high altitude conditions. Although the results did not reach statistical significance, the consistent direction of effect across all included studies supports the pathophysiological plausibility of the antioxidant approach. The methodological limitations identified, including the small number of clinical trials, reduced sample sizes, heterogeneity in interventions, ascent protocols and lack PK/PD analysis limit the strength of the available evidence. Future research should focus on well-designed clinical trials with standardized strategies, controlled exposure protocols, and validated redox biomarkers to more accurately determine the clinical efficacy of antioxidant treatments as a preventive strategy for AMS in populations exposed to high altitude.

## Data Availability

The original contributions presented in the study are included in the article/Supplementary Material, further inquiries can be directed to the corresponding author.

## Nomenclature

RR: Relative Risk
REML: Restricted Maximum Likelihood
CI: Confidence Interval
Q Test: Cochran's Q Test
**τ** ^**2**^: Tau squared, between study variance in metaanalysis
**I** ^**2**^: Heterogeneity Index
WMS: Wilderness Medical Society
RIPC: Remote Ischemic Preconditioning
BID: “bis in die (latín)”, twice a day
CDC: Centers for Disease Control and Prevention EEUU
Nox2: NADPH oxidase 2
GPx: Glutathione Peroxidase
ONSD: Optic Nerve Sheath Diameter
TAC: Total Antioxidant Capacity
TBARS: Thiobarbituric Acid-reactive Substances
8-isoPGF2α: 8-isoprostanes
bpm: beats per minute
PSA: Prostate-Specific Antigen
Hb: Hemoglobin
NS: no significant difference (NDS)
SBP: Systolic Blood Pressure
DBP: Diastolic Blood Pressure
MAP: Mean Arterial Pressure
pCO2: Capillary blood gases
NC: No Changes
PRDX6: Peroxiredoxina 6
GPx3: Glutatión peroxidasa 3
QSOX1: Sulfhidril oxidasa 1
KLKB1: Plasma Kallikrein
VTNC: Vitronectina
F13A: Factor XIII
CHLE: Cholinesterasa
CO8A: Complement C8
u: Unidad adimensional
EPO: Eritropoyetina
TVPG: Transvalvular Pressure Gradient
EBC: Everest Base Camp
NB: Namche Bazaar
cGMP: Cyclic Guanosine Monophosphate
LOOH: Lipid Hydroperoxides
RSNO: S-nitrosothiols
RBC-NO: Red Blood Cell-bound Nitric Oxide
NO: Nitric Oxide
3-NT: 3-Nitrotyrosine
PASP: Pulmonary Artery Systolic Pressure
A: Ascorbate Radical
HR: Heart Rate
OPN: Osteopontina
SOD: Superoxide Dismutase
TST: Total Sleep Time
AHI: Apnea-Hipopnea Index
REM: Rapid Eye Movement Sleep
NREM: NonRapid Eye Movement Sleep
N3: Time spent in deep sleep/Tiempo dedicado al sueño profundo
WAKE: Wake After sleep onset
BR: Breath Rate
NGAL: Neutrophil gelatinase-associated lipocalin; hsCRP: high-sensitivity C-reactive protein
AMS+: develops the pathology
AMS-: does not develop it
HVR: Hypoxic Ventilatory Response
Fol1: Early Follicular Phase
Fol2: Late Follicular Phase
Lut3: Mid-Luteal Phase
VE: Minute Ventilation
